# Underwater Rescue Target Detection Based on Acoustic Images

**DOI:** 10.3390/s24061780

**Published:** 2024-03-10

**Authors:** Sufeng Hu, Tao Liu

**Affiliations:** 1 School of Instrument Science and Engineering, Southeast University, Nanjing 210000, China; 2 China Special Equipment Testing and Research Institute, Beijing 100000, China; 3 Science and Technology on Underwater Vehicle Laboratory, Harbin Engineering University, Harbin 150001, China; gdcptao@hrbeu.edu.cn

**Keywords:** underwater acoustic rescue target detection, deep learning, deep migration learning, acoustic small target detection, lightweight network

## Abstract

In order to effectively respond to floods and water emergencies that result in the drowning of missing persons, timely and effective search and rescue is a very critical step in underwater rescue. Due to the complex underwater environment and low visibility, unmanned underwater vehicles (UUVs) with sonar are more efficient than traditional manual search and rescue methods to conduct active searches using deep learning algorithms. In this paper, we constructed a sound-based rescue target dataset that encompasses both the source and target domains using deep transfer learning techniques. For the underwater acoustic rescue target detection of small targets, which lack image feature accuracy, this paper proposes a two-branch convolution module and improves the YOLOv5s algorithm model to design an acoustic rescue small target detection algorithm model. For an underwater rescue target dataset based on acoustic images with a small sample acoustic dataset, a direct fine-tuning using optical image pre-training lacks cross-domain adaptability due to the different statistical properties of optical and acoustic images. This paper therefore proposes a heterogeneous information hierarchical migration learning method. For the false detection of acoustic rescue targets in a complex underwater background, the network layer is frozen during the hierarchical migration of heterogeneous information to improve the detection accuracy. In addition, in order to be more applicable to the embedded devices carried by underwater UAVs, an underwater acoustic rescue target detection algorithm based on ShuffleNetv2 is proposed to improve the two-branch convolutional module and the backbone network of YOLOv5s algorithm, and to create a lightweight model based on hierarchical migration of heterogeneous information. Through extensive comparative experiments conducted on various acoustic images, we have thoroughly validated the feasibility and effectiveness of our method. Our approach has demonstrated state-of-the-art performance in underwater search and rescue target detection tasks.

## 1. Introduction

With the robust advancement of water transportation, marine resource exploitation, offshore operations, and the underwater entertainment industry, the frequency of accidents has also increased significantly, often resulting from natural disasters like floods and tsunamis. Consequently, the urgent need for rapid underwater rescue operations has become paramount. However, underwater search and rescue missions are inherently challenging and hazardous. Autonomous Underwater Vehicles (AUVs) offer a promising alternative to effectively carry out the search and positioning of underwater rescue targets.

The primary detection methods employed by underwater AUVs are optical and acoustic. However, given that light is significantly attenuated underwater, optical detection is primarily suitable for use within a limited underwater range [1]. Currently, sonar technology has demonstrated effective results in fish searches, wreck detection, underwater geomorphology mapping, and underwater mineral exploration [2]. Nevertheless, its detection capability for weaker targets, such as missing persons, remains inadequate. Furthermore, the scarcity of authentic underwater acoustic rescue target datasets poses a considerable challenge in training machine learning-based detection methods.

In order to make AUVs improve the accuracy of rescue target recognition, this paper targets acoustic rescue small sample training as well as small weak acoustic rescue targets. Due to the scarcity of real underwater acoustic rescue target datasets, this poses a greater challenge to model training. Migration learning [3] has long been applied in machine learning methods in different fields and can effectively alleviate the pressure of a lack of datasets. Valdenegro-Toro M. et al. [4] studied and analyzed migration learning, finding that it can effectively improve model recognition when there is a lack of sonar image datasets. Mckay J. et al. [5] conducted a research study related to the automatic detection of acoustic images of mine-like objects, and in order to solve the problem of the lack of acoustic image datasets, the use of migration learning methods, and the combination of Convolutional Neural Networks (CNN) and Support Vector Machine (SVM), was proposed. Ye et al. [6] attempted to classify underwater targets in side-scan sonar images using pre-trained VGG11 and ResNet18 and proposed a training sample pre-processing method with better implications for the migration learning effect. The transformation from low-complexity tracking raymaps to real sonar images is learned by training GAN (Generative Adversarial Networks) to randomly generate sample data with a consistent distribution of the training dataset [7,8,9,10,11,12,13,14]. Zhang Wenwu et al. [14] used a GAN model and a CycleGAN model to produce sonar images directly from noisy data in order to expand the acoustic image dataset, and the generated results were not good due to poor image data. Huo et al. [15] proposed a semi-synthetic data method mainly for extracting target contours of optical images of ships and aircraft, using a Weibull probability distribution function for the sonar images of Barngrover et al. [11], who preprocessed the optical images and combined them with sonar image features to generate mine-like semisynthetic training data to augment the data set. The complex characteristics of sonar images, such as blurred edges, strong noise, and diverse target shapes, make the data difficult to process, and the above is mainly for objects that are large and distinguishable from their contours and surroundings, such as wrecks, crashed aircraft, and mines. The generation of semi-synthetic pseudo-data is performed, which is not only tedious in the generation process, but also not ideal for improving the detection of drowning victims for underwater rescue targets in real environments. Cheng et al. [16] proposed a multi-domain collaborative migration learning for side-scan sonar images of sunken ships and wrecked aircraft in response to the shortcomings of semi-synthetic data and the inability of pre-trained models based on large optical datasets to match sonar image feature classification, which mainly considers convolutional layers near the input layer to improve the ability to extract low-level edge features from the noisy background of targets in sonar images, which usually have noise-statistical features similar to synthetic aperture radar (SAR) images. Several fully connected layers near the output layer enhance the ability to map high-level feature vectors to the semantic space of sample classes, and optical images with a similar shape as the same target class as sonar features. Chen et al. [17] embedded a multi-head self-attention mechanism into the acoustic target detection network, and captured poor acoustic target features by establishing a global dependency to improve the accuracy of target detection. Li et al. [18] proposed a transformer feature fusion network based on the transformer stack structure to promote information fusion. Moreover, they also propose a novel method called ellipse quality evaluation to improve the reliability of the localization quality estimation, and reduced the false detection rate caused by low resolution. The sonar images of drowning people targeted in this paper are not as clearly distinguished from the target shapes of shipwrecks and wrecked aircraft sonar images, as well as from the surrounding environment, so the mere consideration of high-level shape features is not applicable to drowning people sonar image target recognition.

Underwater acoustic rescue target detection is small target detection, and small targets do not have richer image features like medium and large targets, and the image features of small targets are more difficult to be fully extracted by the network model, so small target detection is vulnerable to interference from noise, background, etc., which makes the network model unable to accurately detect and locate small targets [19]. To solve the problem of the poor performance of small target detection in underwater sonar images, Wang et al. [20] constructed a multi-branch shuttle network and embedded it into YOLOv5s, and replaced the neck of YOLOv5s with BiFPN to detect and identify small targets such as plastic bags, fishing nets, and clothes in sonar images, and verified that the deeper network in the YOLOv5 family is not suitable for the small and weak targets designed in this experiment. However, the method requires a high sonar image background, and the recognition results are often unsatisfactory if the image background is complex and there are more interferences. Yu et al. [21] found that the scale mismatch between the target size of the dataset used for pre-training and the target size of the dataset used for target detection through the detection and recognition experiments on the Tinyperson small target human dataset causes feature representation and detector performance degradation. However, due to the complex background environment of real underwater acoustic images, the scale mismatch between the dataset used for migration training and the dataset used for detector learning can cause false detection of the surroundings of underwater acoustic images if only the scale mismatch between the dataset used for migration training and the dataset used for detector learning is considered.

To effectively tackle the challenges in underwater search and rescue target detection tasks and achieve the visual and auditory detection, recognition, and localization of submerged missing persons, this paper makes the following primary contributions:

1. An automatic underwater acoustic rescue target detection method combining hetero-geneous information hierarchical migration learning for the lack of a underwater acoustic rescue target dataset and a two-way branching convolution module for un-derwater acoustic rescue targets as small targets is proposed. The heterogeneous hierarchical migration learning method utilizes the SAR dataset as well as the optical small target human dataset on land with cross-similar features to the underwater acoustic rescue target dataset for hierarchical migration.
2. The YOLOv5s network model is improved in combination with the ShuffleNetv2 [22,23,24,25] network module to make the method more applicable to underwater unmanned robotic embedded devices for underwater acoustic rescue target detection. The code will be available at https://github.com/Tao-GCPD/Underwater-rescue-target-detection-based-on-acoustic-image (accessed on 8 February 2024).

## 2. Materials and Methods

### 2.1. Lightweight Underwater Acoustic Rescue Target Detection Algorithm

To reduce the storage requirements and improve the detection speed of YOLOV5s on embedded AUV devices, this paper introduces a novel approach. It utilizes a double branch convolutional network module (DbConv) based on the residual network module [26], widening convolutional layers to enhance feature extraction and improve detection accuracy for small acoustic targets while minimizing model parameters. Additionally, the ShuffleNetv2 network module is integrated into YOLOv5s, effectively preserving the detection accuracy for weak targets in acoustic images while significantly reducing computational requirements.

#### 2.1.1. Db-YOLOv5s Architecture and Db-ShuffleNetv2-YOLOv5s Architecture

**Figure 1:**
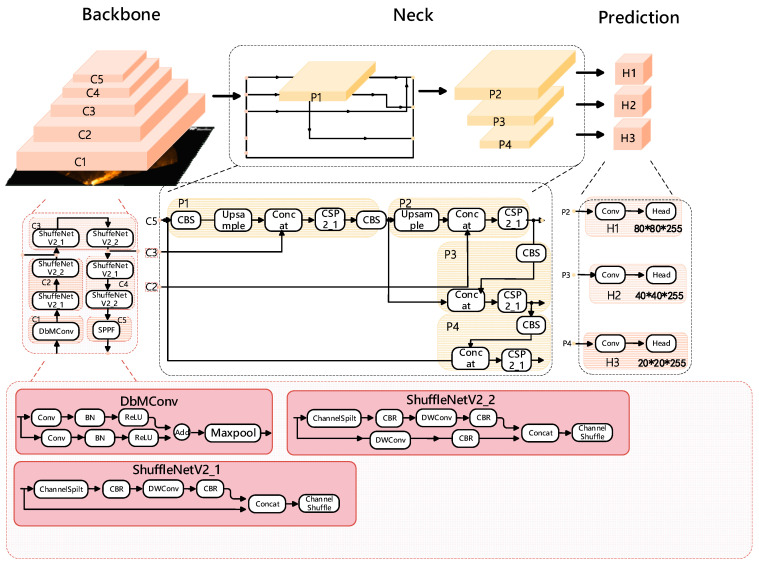
ShuffleNetv2-Db-YOLOv5 s network model structure.

We have modified the YOLOv5s backbone network with ShuffleNetv2 and DbConv modules, as depicted in Figure 1, which includes ShuffleNetV2_1, ShuffleNetV2_2, and SPPF. ShuffleNetV2_1 omits downsampling (Figure 1, while ShuffleNetV2_2 incorporates downsampling (Figure 1). In place of the original YOLOv5s layer 0 network, we have integrated the DbConv module, and for detecting acoustic small targets, we have replaced the backbone network with a combined downsampling module from ShuffleNetV2_1 and ShuffleNetV2_2. This reduces the depth of the backbone network, changing the location of network layers for feature fusion with the Neck network. Specifically, we now perform feature fusion with the layer 2 and layer 4 network image features of the backbone network, making the fusion process shallower.

#### 2.1.2. Double Branch YOLOv5

The method we designed achieves a balance between model lightweighting and accuracy by minimizing the network depth as much as possible. The double branch convolution (DbConv) module, designed for YOLOv5s, is depicted in Figure 1. In Figure 1, the double branch convolution module splits the input feature map into two equally sized branches along the channel dimension. Each branch undergoes the same operations, starting with a 1 × 1 convolution, followed by batch normalization (BatchNorm), ReLU activation, and, finally, their feature maps are merged using addition. This module is primarily intended for small object detection in acoustic rescue scenarios with YOLOv5. It replaces the CBS module in the 0th layer of the YOLOv5s model with smaller convolution kernels, resulting in a model called Db-YOLOv5s.

#### 2.1.3. ShuffleNetv2 Network Module

While large CNN networks have significantly improved image detection and recognition accuracy, in certain specialized scenarios, large and complex models can slow down detection speed, particularly in resource-constrained scenarios such as underwater unmanned vehicles with limited storage and computational resources and high real-time requirements. To address these challenges, lightweight CNN networks like MobileNet and ShuffleNet have been introduced to strike a balance between real-time performance and accuracy.

ShuffleNetv2 [24], proposed by Kuangwei Technology, outperforms ShuffleNet and MobileNetv2 in terms of accuracy. In Figure 1, to alleviate the burden on the YOLOv5s model, we designed double branch convolution modules customized for ShuffleNetv2. It also splits the input feature map into two equally sized branches along the channel dimension. Both branches undergo a 3 × 3 convolution with a stride of 2, followed by batch normalization, ReLU activation, and, finally, their feature maps are merged. The core operation in ShuffleNetv2 is the channel shuffle, which facilitates information exchange between branches.

### 2.2. Heterogeneous Information Hierarchical Transfer Learning

To enhance the detection of underwater acoustic rescue targets, extensive training data is essential. However, our experiment uses a limited underwater rescue target acoustic image dataset, which lacks real underwater images and pertains to small targets. Using only a model-based migration approach, with the optical human dataset from land as the source domain for pre-training and the underwater rescue target acoustic image dataset for fine-tuning, lacks adaptability across domains due to differing statistical properties between optical and acoustic images. Consequently, the trained network becomes overly sensitive to source domain features and struggles with feature extraction in the target domain. To overcome these issues, this paper employs cross-domain similarity features for migration learning, enhancing model training and enabling improved detection performance.

**Figure 2:**
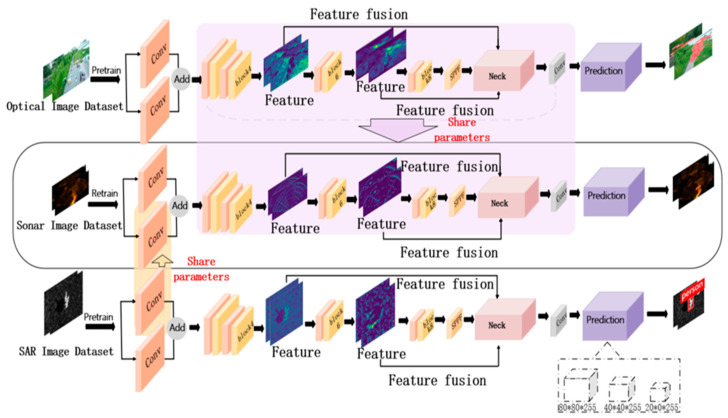
Heterogeneous hierarchical migration learning based on YOLOv5s-DbConv.

Both the small optical dataset and the acoustic dataset share high-level semantic features such as shape and contour, and their target sizes match. For our small-sample acoustic data, we propose a heterogeneous hierarchical transfer learning method based on model migration. We use the heterogeneous hierarchical migration learning method to train the ShuffleNetv2-Db-YOLOv5 model, as illustrated in Figure 2. Initially, we train the SAR dataset and the optical human dataset separately on the Db-YOLOv5s model, saving the trained weight files. We then extract the trained weights from layer 0 in the SAR dataset weight file and migrate them to the corresponding target ShuffleNetv2-Db-YOLOv5 network model for retraining in the target domain. Similarly, we extract and migrate weights from layer 1 to layer 24 from the trained weight file of the optical human dataset to the target Db-YOLOv5s network model for retraining on the target position image dataset.

**Figure 3:**
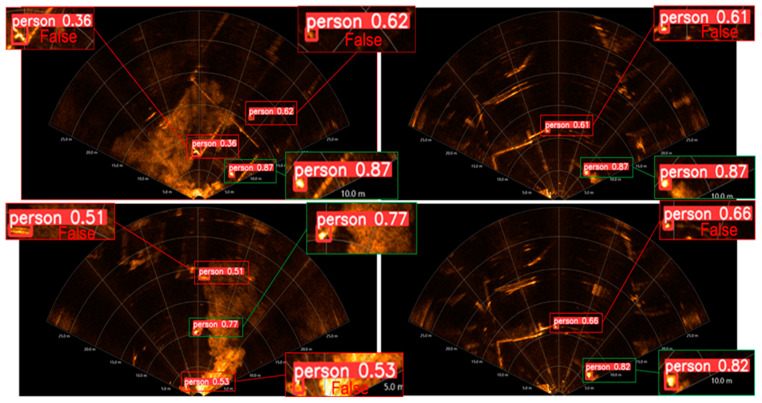
Outdoor complex background underwater rescue target acoustic image detection effect.

When simulating real rescue scenarios, detecting underwater rescue targets acoustically can be challenging due to the complex background in Figure 3. This background includes floating docks, small boats, underwater noise, and other objects, leading to false detections. In the magnified detection results, the green rectangle represents correct detections by the algorithm, while the red rectangle indicates erroneous detections. These errors often occur when strong reflections from dot-like and bar-like objects in the complex underwater background interfere with the detection of underwater acoustic rescue targets.

## 3. Results

### 3.1. Data Set Used

In this paper, the acoustic image-based underwater rescue target image data was acquired in the Qingdao outdoor development sea dock and the Harbin Engineering University comprehensive experimental pool, the experimental equipment was BlueView M900-130 multi-beam imaging sonar, and the multi-beam imaging sonar-related parameters are shown in Table 1.

**Figure 4:**
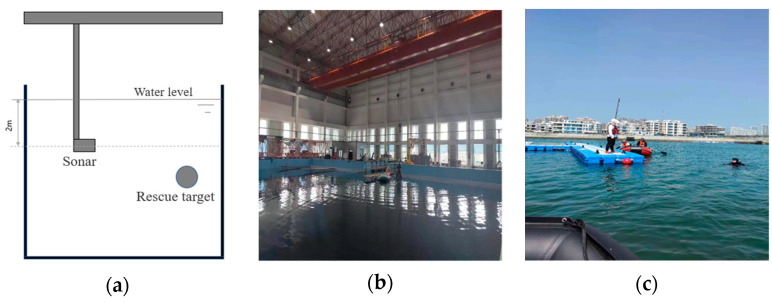
(**a**) Schematic diagram of acoustic image data acquisition for underwater rescue targets, (**b**) The comprehensive experimental pool, and (**c**) Qingdao open sea terminal.

**Figure 5:**
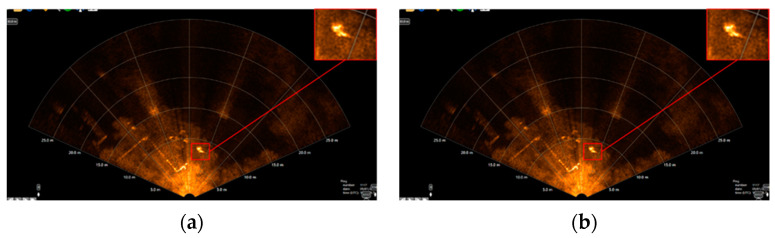
(**a**) Acoustic image of the pool underwater rescue target. (**b**) Acoustic image of outdoor underwater rescue target.

Figure 4a shows a schematic diagram of the acoustic image data acquisition for underwater rescue targets. Figure 4b displays the experiment conducted in the Harbin Engineering University’s comprehensive pool, measuring 50 m in length, 30 m in width, and 10 m in depth. To enable comparative experiments on small target recognition, car tires filled with weights are placed in the pool for filming. Recognizing that the real underwater environment is more complex, with added foreign objects and noise, we conducted data image acquisition in the Qingdao open sea wharf, as shown in Figure 4c. Divers, equipped with diving gear, mimic drowning scenarios in designated areas, capturing underwater rescue target acoustic images. We filmed various sea areas around the wharf, capturing outdoor underwater rescue target acoustic images. Examples of the acquired images is shown in Figure 5. These images are influenced more significantly by their surrounding environment compared to the pool’s underwater rescue target acoustic images. 

The target domain is the underwater rescue target acoustic image dataset, where the training set and test set are divided as shown in Table 2. The purpose of this division is due to the outdoor underwater rescue target acoustic images having a more complex background and noise, being more difficult to detect, and more in line with the real rescue environment, compared to the pool underwater rescue target acoustic images. The training set having a smaller proportion of outdoor underwater rescue target acoustic images and the test set having a larger proportion of outdoor underwater rescue target acoustic images ensure the generalization of the method used in the experiment.

**Figure 6:**
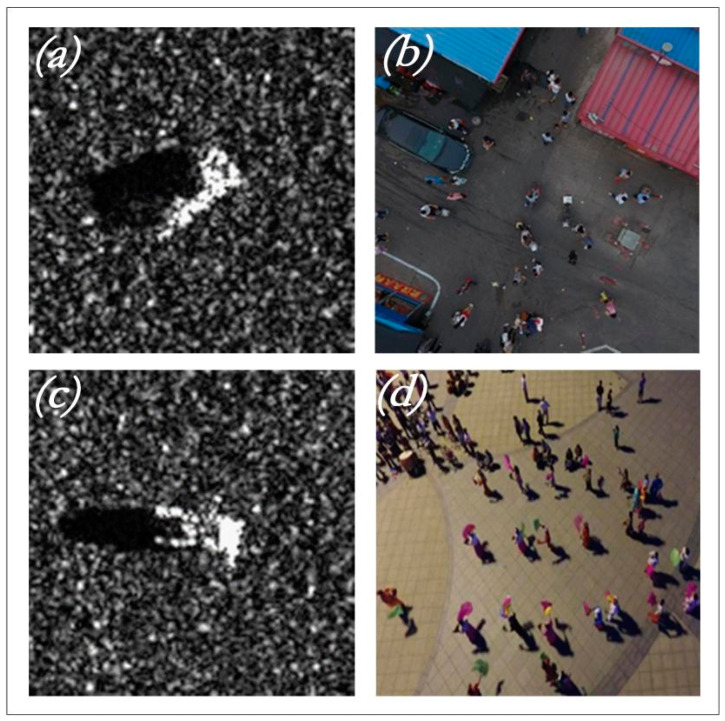
SAR data set and optical human dataset. (**a**,**c**) are the SAR dataset samples. (**b**,**d**) are the optical human dataset samples.

The source domain data required for the heterogeneous information hierarchical migration learning method used in this experiment are the SAR (Synthetic Aperture Radar) dataset, as shown in Figure 6, and the small target human optical image dataset, as shown in Figure 6a,c, The source is divided into data sets as shown in Table 3.

The SAR dataset is derived from the MSTAR program, providing ground-based stationary target data obtained by a high-resolution spot-beam synthetic aperture radar (SAR) sensor with a 0.3 m × 0.3 m subscale. It primarily contains SAR sliced images of stationary vehicles captured at various azimuth angles.

The optical human dataset (Figure 6b,d) on land is constructed from images within the VisDrone2019 dataset, collected by Tianjin University’s AISKYEYE team. This extensive dataset includes over 2.6 million manually annotated frames of common targets like pedestrians, cars, bicycles, and tricycles. The optical human dataset used in this paper specifically comprises pedestrian data from this source.

### 3.2. Evaluation Indicators

In this paper, the evaluation metrics for the detection of underwater acoustic rescue targets are mainly the mean average precision (mAP), as well as the computational volume of forward inference (GFLOPs) and the number of parameters.

*TP* represents correctly identified actual targets with correct predictions, while *FP* denotes incorrect actual targets detected as correct. TN indicates correct actual targets not detected, and *FN* represents incorrect actual targets not detected. Detection is considered correct (*TP*) if the intersection ratio of the true frame and the predicted frame exceeds the set threshold; conversely, it is deemed incorrect (*FN*) if it falls below the threshold.

(1) $$\mathrm{Precision}=\frac{TP}{TP+FP}$$

The accuracy rate, as defined by Formula (1), quantifies the ratio of correctly detected targets (*TP*) to the total targets detected as correct. Notably, a higher number of correctly identified actual targets leads to a higher accuracy rate when a certain number of samples yield correct predictions.

(2) $$\mathrm{Recall}=\frac{TP}{TP+FN}$$

The recall rate, expressed in Formula (2), quantifies the ratio of correctly detected targets (*TP*) to the total actual targets detected. When the number of samples correctly identifying actual targets remains constant, a larger number of samples correctly predicted as targets results in a higher recall rate. This implies that a higher recall rate indicates fewer missed correct targets in the network model’s predictions, ultimately yielding more accurate results.

### 3.3. Experimental Environment

The following is the host environment information used in the experiments: the operating system was Ubuntu 18.04, the programming language was Python 3.7, the GPU model was Nvidia RTX 3090 produced by Nvidia, Santa Clara, CA, USA, and the deep learning framework was PyTorch 1.8.1. To accelerate the training of neural network models, we also installed CUDA and CuDNN. More detailed environmental information is provided in Table 4.

### 3.4. Performance Analysis

In this paper, we experimented with the Db-YOLOv5s model, freezing different combinations of network module weight parameters; namely, layer 0, layers 0 and 1, layers 0 to 3, and layers 1 to 3. The results are summarized in Table 5. Comparing the outcomes to not freezing any network module weight parameters, we observed that freezing layer 0 and layers 1 to 3 enhanced the model’s mAP for underwater rescue acoustic target detection. Notably, freezing layer 0 provided the most significant improvement, raising the mAP by 0.013 compared to not freezing any layer. This improvement is attributed to the freezing of weight parameters for both the SAR dataset training migration and the optical small-target human dataset migration, which subsequently preserves and reinforces cross-similarity between datasets. Fine-tuning the remaining unfrozen layers with the underwater rescue target acoustic image dataset further enhances detection accuracy. This underscores the feasibility of the proposed heterogeneous information hierarchical migration learning principle.

We used a training strategy that involved pre-training an acoustic image dataset with an optical image dataset to compare Db-YOLOv5s, our improved small target detection algorithm, with the standard YOLOv5s algorithm. Then, we introduced a proposed heterogeneous information hierarchical transfer learning strategy to train the Db-YOLOv5s model. Finally, we conducted a comparison experiment to address background false detections in acoustic images of underwater rescue targets using the same strategy. We evaluated different methods, including freezing various layer network weight parameters, and also trained the improved lightweight model, Db-ShuffleNetv2-YOLOv5s, using a similar approach. The results of these experiments are presented in Table 5.

**Figure 7:**
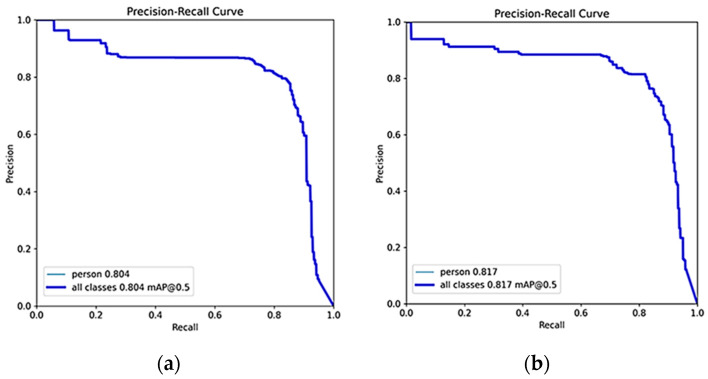
P–R curves for freezing different layers of YOLOv5s-DbConv model based on heterogeneous hierarchical migration learning. (**a**) Original unfrozen network layer and (**b**) Freeze layer 0.

**Figure 8:**
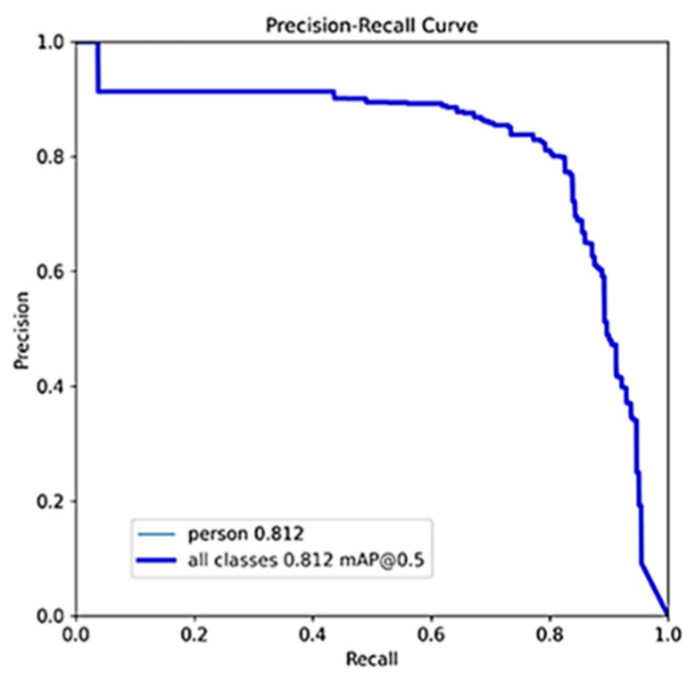
P–R plot of ShuffleNetv2-YOLOv5s-DbConv algorithm based on heterogeneous hierarchical migration learning freezing layer 0.

In Table 5, “OP/AF” denotes optical image pre-training and acoustic image fine-tuning, while “HTL” represents hierarchical transfer learning using heterogeneous information. Compared to the original YOLOv5s algorithm, our Db-YOLOv5s model, designed for acoustic small target detection in underwater rescue scenarios, outperforms in terms of mAP and slightly surpasses it in parameter count. However, it substantially exceeds the original YOLOv5s model in terms of computational complexity (GFLOPs). Using our proposed heterogeneous information hierarchical migration learning for training, Db-YOLOv5s yields a noteworthy mAP improvement of 0.03 compared to the strategy of pre-training the acoustic image dataset with optical images. The precision–recall (P–R) plot for this approach is depicted in Figure 7a. Freezing the layer 0 network module weight parameters and freezing network module weight parameters for layers 1, 2, and 3 both enhance the mAP for underwater rescue acoustic target detection compared to not freezing any layers. Notably, freezing the layer 0 parameters yields the most substantial improvement, with an mAP increase of 0.013 compared to not freezing any layer, as shown in Figure 7b. The improved lightweight model, ShuffleNetv2-YOLOv5s-DbMConv, proposed in this paper, significantly reduces the computational complexity (GFLOPs) and parameter count at the expense of a slightly lower mAP compared to the Db-YOLOv5s model. Its P–R plot is presented in Figure 8.

**Figure 9:**
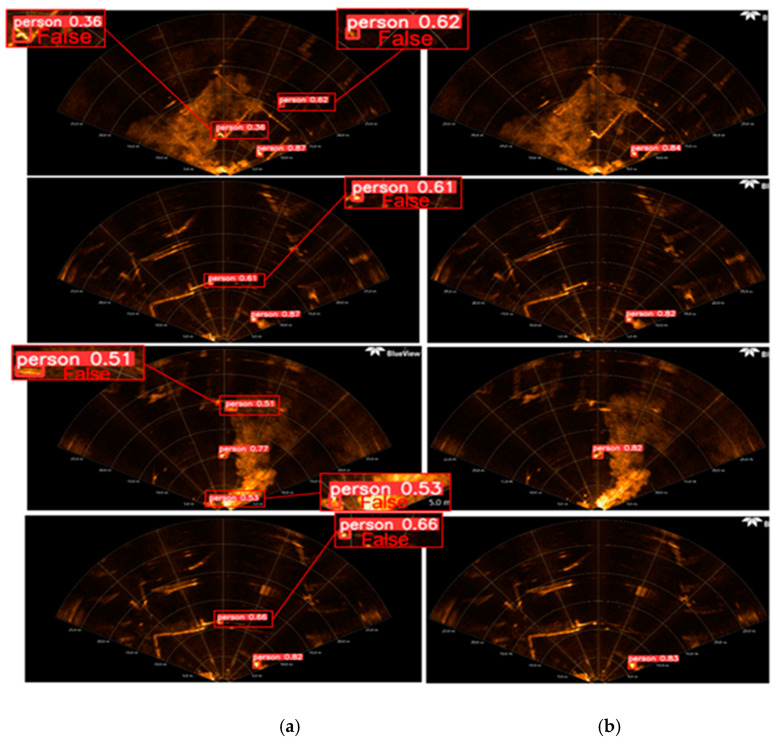
Comparison of the detection effect of the proposed method on the complex background of underwater acoustics. (**a**) False detection and (**b**) Correct detection.

The described method involves heterogeneous hierarchical migration learning, initially migrating source domain dataset weight parameters to the target network model and freezing layer 0 network module weight parameters. Subsequently, a retraining fine-tuning strategy is employed using the target domain dataset to detect the images with false detections in Figure 9b. The comparison of detection results is illustrated in Figure 9a, where Figure 9b highlights the presence of false detections, and Figure 9 demonstrates the effect of freezing the weight parameters of the first layer network module. This training strategy effectively reduces false detections in the background when detecting underwater rescue target acoustic images.

The training of the Db-ShuffleNetv2-YOLOv5s model, using heterogeneous hierarchical transfer learning with a frozen layer 0 network module, involves 300 epochs with a learning rate of 0.01 and an SGD optimizer for network gradient updates. To assess the detection performance of our algorithms on underwater rescue target acoustic image datasets, we compare them with commonly used YOLOv3 and SSD target detection algorithms, as well as the YOLOv5s model trained with official pre-training weights as the source domain. The comparative results under the same experimental settings are summarized in Table 6. It is evident that our proposed method in this paper outperforms other models in terms of mAP for underwater rescue target detection in acoustic images.

### 3.5. Ablation Experiments

We compared the metrics mAP, GFLOPs, and Parameters for four cases: original YOLOv5s, Db-YOLOv5s, ShuffleNetv2-YOLOv5s, and Db-ShuffleNetv2-YOLOv5s, using YOLOv5s as the base model. The results are summarized in Table 7.

According to Table 7, the Db-YOLOv5s model achieves the highest mAP. The lightweight Db-ShuffleNetv2-YOLOv5s improved model follows closely, with only a slight 0.05 mAP difference but with significantly better GFLOPs and parameter metrics compared to Db-YOLOv5s. The Db-ShuffleNetv2-YOLOv5s model also improves mAP compared to the ShuffleNetv2-YOLOv5s model, albeit with minor increases in GFLOPs and parameters. The lightweight ShuffleNetv2-YOLOv5s-DbConv improved model outperforms the original YOLOv5s network model in terms of mAP, GFLOPs, and parameters. Therefore, our proposed lightweight ShuffleNetv2-YOLOv5s-DbMConv model sacrifices a slight mAP accuracy for a significant reduction in GFLOPs and parameters.

## 4. Discussion

The main operation scene of our proposed underwater rescue target detection and recognition algorithm based on acoustic images is single rescue target detection in complex scenes, and the rescue target is in the underwater suspension state. However, in an actual underwater rescue situation, there are multiple rescue targets and the rescue target is sinking. In future research, more attention should be given to the detection in this case, including the addition of 3D detection. The hierarchical transfer learning method of heterogeneous information based on model transfer proposed in this paper needs to train the SAR data set and the optical small target data set in the source domain and the underwater acoustic rescue target data set in the target domain, respectively, during training, which will consume more time. In subsequent research, we will further optimize the training strategy to reduce the training time.

## 5. Conclusions

This paper introduces an innovative framework for the underwater rescue target detection and recognition in acoustic images, tailored to address the unique challenges of complex underwater rescue scenarios. To enhance the detection accuracy, a hierarchical migration learning method is proposed, leveraging enhanced YOLOv5s with heterogeneous information. This approach significantly boosts accuracy by leveraging cross-similar features from SAR images, optically small target human images, and underwater acoustic rescue target images, addressing issues related to small dataset sizes and cross-domain adaptability. To optimize for the real-time deployment on resource-constrained devices, a two-branch convolutional module combined with ShuffleNetv2 is introduced, reducing computational requirements and model parameters, while maintaining detection accuracy. Freeze-and-train strategies are employed to further improve the detection accuracy in complex underwater settings. Comparative experiments on underwater acoustic tires and underwater acoustic rescue target datasets demonstrate the method’s ability to correctly identify these types of underwater acoustic small targets, outperforming traditional detection methods. Overall, this work represents a significant step forward in underwater rescue target detection, promising to improve the safety and efficiency of underwater rescue operations in real-world scenarios.

## Figures and Tables

**Table 1:** M900-130 multi-beam imaging sonar parameters table.

Parameter Name	Maximum Range	Resolution	Frequency	Maximum Viewing Angle	Beamwidth
Numerical value	100 m	1.3 cm	900 kHz	130°	1 × 20°

**Table 2:** Training set and test set division of the target domain data set.

Acoustic Image	Training Set	Test Set
Rescue targets outdoor	17	192
Rescue targets in pools	1270	51

**Table 3:** Training set and test set division of the source domain dataset.

	Training Set	Test Set
SAR data set	4581	591
Pptical human dataset	5471	648

**Table 4:** Experimental environment configuration.

Operating System	Ubuntu 18.04
Programming Languages	Python 3.7
Deep Learning Framework	PyTorch 1.8.1
Other Environments	CUDA11.1, cudnn8.0
CPU Model	Intel i9 9900 K 3.6 GHz × 16
GPU Model	RTX3090 24 G
RAM	32 G

**Table 5:** Comparison of the effects of different methods.

Model	Method	Freeze	mAP	GFLOPs	Parameters
YOLOv5s	OP/AF		0.732	15.8	7012,822
Db-YOLOv5s	OP/AF		0.774	60.4	7009,590
Db-YOLOv5s	HTL		0.804	60.4	7009,590
Db-YOLOv5s	HTL	Level 0	0.817	60.4	7009,590
Db-YOLOv5s	HTL	Level 0–1	0.745	60.4	7009,590
Db-YOLOv5s	HTL	Level 0–3	0.775	60.4	7009,590
Db-YOLOv5s	HTL	Level 1–3	0.809	60.4	7009,590
MobileNetv3-YOLOv5s	HTL		0.724	6.1	3524,708
Db-ShuffleNetv2-YOLOv5s	HTL	Level 0	0.812	5.9	3178,090

**Table 6:** The comparative results.

Method	mAP
YOLOv3	0.727
BotNet-FPN	0.751
YOLOv4	0.742
SSD(MobileNetv2)	0.458
YOLOv5s (official pre-training weights)	0.768
This paper’s proposed method	0.812

**Table 7:** Comparison of the effects of different methods.

	DbConv	ShuffleNetv2	mAP	GFLOPs	Parameters
1	-	-	0.735	15.8	7,012,822
2	✓	-	0.817	60.4	7,009,590
3	-	✓	0.786	5.8	3,177,626
4	✓	✓	0.812	5.9	3,178,090

## Data Availability

The data presented in this study are available on request from the corresponding author.
